# Recent advances in glucocorticoid regulation of bone and the bone marrow niche: Genetic and pharmacological approaches to understand and prevent bone loss

**DOI:** 10.1016/j.coemr.2026.100617

**Published:** 2026-06-16

**Authors:** Isabel D. Hermsmeyer, Ziru Li, Ormond A. MacDougald

**Affiliations:** 1 University of Michigan, Department of Anthropology, Ann Arbor, MI, USA; 2 MaineHealth Institute for Research, Scarborough, ME, USA; 3 University of Michigan Medical School, Department of Molecular & Integrative Physiology, Ann Arbor, MI, USA; 4 University of Michigan Medical School, Department of Internal Medicine, Ann Arbor, MI, USA

## Abstract

Glucocorticoids are essential steroid hormones whose excess – whether from therapeutic use or endogenous overproduction – causes significant bone loss and fracture risk. In bone, glucocorticoids act directly on osteoblasts, osteocytes, and osteoclasts via the glucocorticoid receptor and indirectly through bone marrow adipose tissue and shared mesenchymal progenitors. An important mechanism involves suppression of canonical Wnt/β-catenin signaling, which reduces osteogenesis and increases marrow adiposity. Several pharmacological and plant-derived compounds with therapeutic promise target Wnt and other glucocorticoid-regulated signaling pathways. Emerging evidence highlights intermediary and potential therapeutic roles for niche-derived extracellular vesicles in bone loss associated with glucocorticoids. Comparing results between studies is complicated by underreporting of environmental and biological variables that may interact with glucocorticoids to affect bone or the marrow niche, which underscores the need for greater methodological rigor in preclinical studies.

## Endogenous and exogenous glucocorticoids impact bone

**Figure 1:**
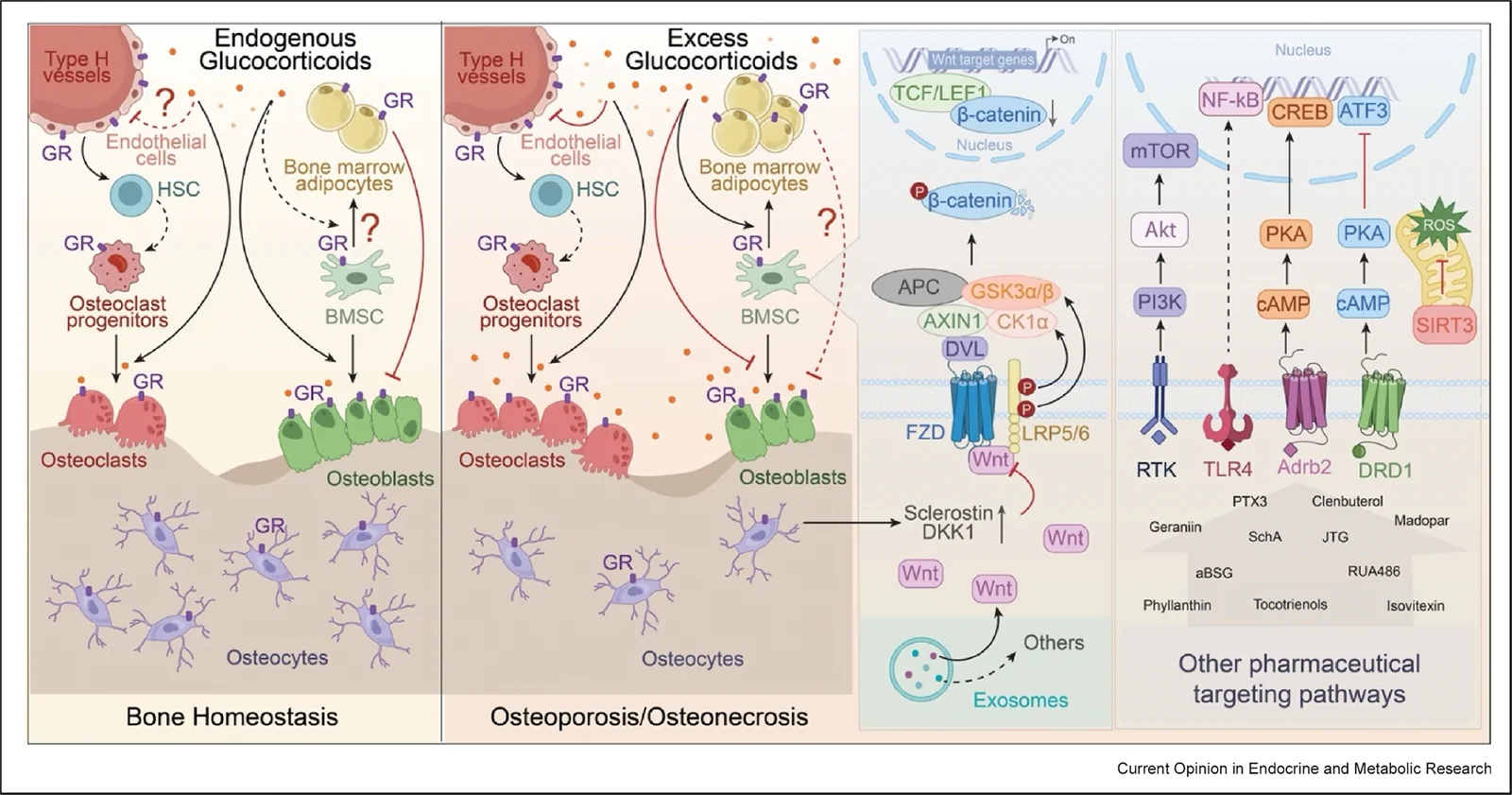
Overview of mechanisms underlying physiological and pathological effects of glucocorticoids on bone. Highlighted are causal and therapeutic roles of Wnt signaling. A variety of pharmaceuticals target Wnt and other signaling pathways to combat glucocorticoid-induced bone loss.

Glucocorticoids are steroid hormones synthesized in the adrenal cortex from cholesterol through the action of enzymes regulated by the hypothalamic–pituitary–adrenal axis. They play important roles in metabolic homeostasis by mobilizing lipids and amino acids during fasting, promoting gluconeogenesis, and reducing insulin sensitivity. Endogenous glucocorticoids also play a positive role in maintaining bone mass and homeostasis. Glucocorticoids exert potent anti-inflammatory and immunosuppressive actions by suppressing cytokine production and immune cell activation, which has made glucocorticoid agonists indispensable for many clinical indications. Unfortunately, exogenous glucocorticoid therapy, as well as endogenous cortisol excess, is among the most common threats to skeletal integrity. Even modest, sustained elevations in circulating glucocorticoids lead to rapid bone loss, deterioration of microarchitecture, and disproportionate increases in fracture risk, defining glucocorticoid-induced osteoporosis as a distinct metabolic bone disease [1]. In bone, glucocorticoids act directly on osteoblasts, osteocytes, and osteoclasts via the glucocorticoid receptor (GR) to suppress bone formation, alter remodeling dynamics, and promote cell death. In addition, their skeletal impact is amplified by indirect effects on bone marrow adipocytes (BMAds) and shared mesenchymal progenitors that shift lineage allocation toward adipogenesis [2,3]. Glucocorticoids mediate these actions through their regulation of intercellular signaling pathways, including inhibition of canonical Wnt signaling and upregulation of Wnt antagonists such as DKK1 and sclerostin, as well as secreted factors such as FGF23, GDF11, and exosomes, among others that influence both local bone turnover and systemic metabolism [4–7]. Thus, elevated glucocorticoids rapidly remodel this network of direct receptor-mediated actions and indirect niche-derived signals to cause a glucocorticoid-induced osteoporosis (Figure 1). Considerable drug discovery efforts have been directed toward pathway-selective strategies and mining of traditional medicines to combat deleterious glucocorticoid-regulated signaling networks, including downstream effects on Wnt signaling [8,9], and cytokine and apoptotic pathways [10,11], without compromising the anti-inflammatory benefits of glucocorticoid therapy (Table 1).

## Mechanisms by which glucocorticoids regulate bone mass, including potential roles of bone marrow adipose tissue

Glucocorticoids exert their complex and sometimes opposite effects on bone mass through multi-factorial mechanisms, including direct mechanisms on bone cells and indirect effects mediated by other cell types, including BMAds, which are now recognized as a critical endocrine and paracrine regulators of skeletal homeostasis (Figure 1; [12–14]. How endogenous glucocorticoids regulate bone mass and bone marrow adipose tissue (BMAT) in vivo has been difficult to parse, and results are dependent on the model and approach and are undoubtedly affected by environmental context, which is often poorly documented in the literature (Table 2). Whereas under physiological conditions, endogenous glucocorticoids in mice exert an anabolic effect on osteoblasts and bone, elevated glucocorticoid signaling has a strong catabolic effect. For example, mouse models where endogenous glucocorticoid metabolism is reduced by GR knockout, overexpression of 11β-HSD2/knockout of 11β-HSD1 to decrease local corticosterone concentrations, have generally suggested that endogenous glucocorticoid signaling is required for bone development and to maintain bone mass and structure under standard conditions (Table 2; Reviewed in Ref. [2]). However, numerous studies also find that under basal or caloric restricted conditions, transgenic mice with low glucocorticoid signaling do not have altered bone variables [15,16], although with aging or a high-fat diet [17,18], protection against bone loss is observed. Discrepancies between studies suggest that the effects on bone of reducing endogenous corticosterone, the active murine glucocorticoid, are impacted by unknown experimental, environmental, or physiological variables.

In contrast, high-dose exogenous glucocorticoids, such as corticosterone or prednisolone, profoundly and consistently decrease bone mass. They directly target osteoblasts and osteocytes via the GR, leading to reduced osteoblast number, decreased osteoid and bone formation rate, and increased apoptosis of osteoblasts and osteocytes. This suppression of osteoblast activity is a major driver of glucocorticoid-induced bone loss and reduced bone strength, independent of changes in osteoclast number. Mechanistically, exogenous glucocorticoids inhibit osteoblast differentiation and function largely through the monomeric GR (Figure 1), which represses osteoblast-derived cytokines such as IL-11 via tethering to AP-1, thereby attenuating osteoblastogenesis and bone formation [19].

a) Direct effects on osteoblasts and osteoclasts.

A substantial body of genetic evidence demonstrates that glucocorticoid signaling acts directly within osteoblast and osteoclast lineages, where it regulates bone formation, resorption, and marrow adiposity through cell-specific actions of the GR and local glucocorticoid metabolism (Table 2). Transgenic overexpression of *Hsd11b2* in osteocalcin-lineage osteoblasts protects against pharmacologic and age-related bone loss and apoptosis, supporting a detrimental role for canonical glucocorticoid signaling in bone [15,17]. In contrast, *Hsd11b2* overexpression in *Col1a1*-lineage osteoblasts causes mild trabecular loss, and osteoblast-specific deletion of the GR confirms that GR mediates glucocorticoid-induced osteopenia while also being required for normal osteoblast differentiation [20]. Consistent with this dual role, conditional deletion of GR in Runx2-expressing osteoblasts demonstrates that GR mediates glucocorticoid-induced loss of bone mass, as young GR^Runx2–CRE^ mice exhibit decreased spinal bone density due in part to impaired osteoblast differentiation [19]. Targeting GR earlier in the osteoblast lineage with conditional knockout of GR in Osterix-expressing osteoprogenitors and downstream differentiated cells under chronic caloric restriction results in increased BMAT accumulation and bone loss [21], with aging of the same GR^Osx–CRE^ model to 21 months revealing persistent genotype effects of decreased bone and increased BMAT, along with marked alterations to muscle mass and activity [22]. In parallel, osteoblast-specific deletion of 11β-HSD1 produces no baseline skeletal phenotype in male mice but confers resistance to high-fat diet–induced bone loss and metabolic dysfunction, including reduced white adipose tissue and improved glucose tolerance [23]. Direct glucocorticoid signaling in osteoclasts also contributes to skeletal outcomes, as osteoclast-specific GR knockout mice are protected from dexamethasone-induced inhibition of bone formation, despite minimal baseline effects and additional deletion in LysM-expressing immune cells [24]. Glucocorticoids also affect bone loss through receptors other than GR — for example, thinning of cortical bone by prednisolone is mediated, in part, by the mineralocorticoid receptor [25]. In addition, global knockout of Tau, an atypical low-affinity GR, demonstrates that Tau is required for glucocorticoid-stimulated osteoclastogenesis and the development of osteoporosis [26].

b) Indirect effects through BMAT or other cell types.

### BMAds

Beyond direct actions on osteoblasts and osteoclasts, considerable focus has been placed on BMAT as an indirect mediator of glucocorticoid effects (Figure 1). Although the presence of constitutive BMAds within the marrow niche has a net negative effect on bone mass [12], BMAT also serves important functional roles within the marrow environment. For example, BMAd lipolysis supports the function of osteoblasts and the formation of bone in male mice during energy deficits, including caloric restriction, cold exposure, and bone regeneration [13]. BMAds are also required to maintain hematopoietic stem and progenitor cell numbers [12], with lipolysis supporting myelopoiesis during regeneration following irradiation [13]. In addition, emerging evidence indicates that glucocorticoids induce senescence in BMAds, promoting a senescence-associate secretory phenotype (SASP) that impairs osteoblast function and contributes to bone loss [27]. Despite these emerging BMAd-mediated mechanisms, contributions of glucocorticoid signaling specifically within BMAds to skeletal outcomes appear to be limited, as targeted deletion of the GR in this cell population results in only a modest increase in trabecular bone variables in female BMAd-*Nr3c1*^−*/*−^ mice, with no effects observed in males or under caloric restriction [28]. Further, glucocorticoid action on BMAT can be dissociated from skeletal outcomes in a cell type- and sex-specific manner. Whole-body deletion of *Hsd11b1* suppresses caloric restriction–induced bone marrow adiposity in male but not female mice, without detectable effects on bone mass [16]. Earlier work similarly demonstrated that in mice with global loss of *Hsd11b1*, local glucocorticoid activation is required for BMAT accumulation but is not essential for basal osteogenesis [29]. BMAT is a disproportionate source of local and circulating adiponectin, and pharmacologic activation of adiponectin signaling using the adiponectin receptor agonist AdipoRon blocks glucocorticoid-induced accumulation of BMAds without substantially altering glucocorticoid-induced bone loss, reinforcing the concept that BMAT can be selectively modulated independently of skeletal outcomes [30]. More broadly, glucocorticoid signaling within adipose tissues is modulated by sex hormones in a depot-specific manner. In gonadal white adipose tissue, androgens are required for a robust glucocorticoid transcriptional response, and cotreatment with an androgen amplifies corticosterone-driven adiposity. In contrast, interscapular brown adipose tissue shows glucocorticoid-responsive GR target genes even without androgens [31]. Although considerable work remains to delineate mechanistic relationships between BMAT and bone, phenome-wide association studies reveal that genetic predisposition to increased marrow adiposity is positively associated with osteoporosis and fractures, whereas Mendelian randomization unveils a site-specific causal relationship, with femoral, but not spinal marrow adiposity associated with increased osteoporosis and fracture risk [14]. Further work is required to test whether high marrow fat increases the risk of osteoporosis after glucocorticoid (GC) treatment.

### Other cell types in the niche

In addition to BMAds, the marrow niche also contains stromal, vascular, immune, progenitor, and other cell populations that may contribute to skeletal regulation. Through endothelial cells, glucocorticoids impair marrow vascularization, which locally depletes fatty acids, creating an energy-deficient marrow niche that secondarily diminishes both osteoblast and osteoclast activity. GCs and fatty acid metabolism cooperatively program M2c polarization of macrophages, leading to BMP2 secretion, which in turn modulates osteogenesis. Restoring fatty acid supply to macrophages, including via targeted lipid nanoparticles, reprograms their metabolism and partially rescues bone formation and fracture repair [32].

Glucocorticoids reprogram the marrow niche at the level of mesenchymal progenitors, which are typically referred to as bone marrow stromal cells (BMSCs), along with BMAd-lineage cells, to stimulate adipogenesis at the expense of osteogenesis. Recent evidence from glucocorticoid-induced osteonecrosis models demonstrates that high-dose glucocorticoids promote cellular senescence and epigenetically bias BMSCs toward adipogenic differentiation at the expense of osteogenesis, altering the balance between bone and BMAd lineages [33–36]. Consistent with this progenitor-centered framework, early B-cell factor (EBF1) deficiency as the result of Prx-cre produces an adynamic, prematurely aged skeleton with impaired osteogenesis, increased and dysregulated marrow adiposity, and reduced GR signaling in BMSCs [37]. Complementing these findings, prenatal glucocorticoid exposure depletes marrow Adipoq^+^ adipogenic lineage precursors, disrupting redox homeostasis, reducing type H vasculature, and impairing osteoblastogenesis, whereas genetic ablation of these cells recapitulates many of these skeletal defects [38]. Recent work further supports this model, showing that glucocorticoid-induced bone loss reflects coordinated changes in skeletal stem cell lineage allocation and angiogenic crosstalk, with Basigin blockade conferring protection [39].

## Glucocorticoids decrease bone mass through suppression of Wnt/β-catenin signaling

Canonical Wnt/β-catenin signaling is a critical regulator of bone homeostasis, with activation of the pathway leading to increased bone mass and strength. Interestingly, substantial experimental and clinical evidence links exogenous glucocorticoid exposure to bone loss through suppression of canonical Wnt/β-catenin signaling. In osteoblasts and osteocytes, glucocorticoids directly inhibit Wnt signaling, reducing osteoblast differentiation, bone formation, and osteocyte survival; effects that are reversible through estrogen receptor activation or pharmacologic stimulation of downstream pathways that restore Wnt activity, including PKG2 signaling [40,41]. Osteocytes emerge as key mediators of this response, as glucocorticoid exposure in rodents increases osteocyte-derived sclerostin, a potent Wnt antagonist that suppresses osteogenesis while promoting osteoclastogenic signaling [42]. Consistent with these mechanisms, clinical studies in post-menopausal women with glucocorticoid-induced osteoporosis demonstrate strong associations between circulating sclerostin levels and vertebral marrow adiposity, supporting a link between glucocorticoid-mediated Wnt inhibition and altered marrow composition [43]. Therapeutically, neutralization of sclerostin dissociates bone formation from resorption, producing marked gains in bone mass and fracture protection, underscoring the central role of Wnt suppression in glucocorticoid-associated skeletal pathology [4]. In addition, plant-derived molecules such as tocotrienols and schisandrin A inhibit glucocorticoid-induced bone loss through activation of the Wnt signaling pathway [8,9].

Beyond direct effects on osteoblast lineage cells, glucocorticoid-induced suppression of Wnt signaling disrupts the broader marrow niche, integrating skeletal, vascular, and progenitor-level dysfunction. In models of steroid-induced osteonecrosis, glucocorticoids reduce ZEB1 expression in type-H endothelial cells, leading to impaired angiogenesis and osteogenesis through suppression of Wnt/β-catenin signaling, whereas pharmacologic Wnt activation rescues both vascular and skeletal defects [44]. At the progenitor level, glucocorticoid exposure suppresses β-catenin signaling in BMSCs, shifting lineage commitment from osteoblasts toward adipocytes. Moreover, genetic deletion of β-catenin in Col2^+^ mesenchymal progenitors phenocopies the full spectrum of glucocorticoid-induced osteonecrosis, establishing Wnt suppression as a causal driver of marrow adiposity and collapse of the femoral head [45].

## Extracellular vesicles as mediators of Wnt signaling and bone repair under glucocorticoid stress

Emerging evidence indicates that extracellular vesicles (EVs) constitute an important mechanism through which Wnt signaling is propagated across tissues and restored in glucocorticoid-compromised bone (Figure 1). In adipose tissue, canonical Wnt/β-catenin signaling plays a central role in regulating lipid metabolism and multicellular niche homeostasis, and Wnt-related signals can be transferred between cell types via EV-mediated cargo, highlighting a broader intercellular Wnt communication network extending beyond cell-autonomous signaling [46,47]. Within the skeletal niche, EVs similarly act as vehicles for Wnt pathway modulation under glucocorticoid stress: exosomes derived from mechanically-stimulated BMSC reverse dexamethasone-induced suppression of osteoblast proliferation and differentiation by reactivating canonical Wnt/β-catenin signaling, an effect partially abrogated by Wnt inhibition [48]. Consistent with this mechanism, lithium enhances osteogenesis in glucocorticoid-induced osteonecrosis models by increasing the secretion of BMSC-derived exosomal Wnt10a, thereby activating β-catenin signaling and promoting bone regeneration [49]. Beyond endogenous vesicle signaling, biomimetic and engineered EV platforms have further demonstrated the capacity to restore osteogenesis and angiogenesis in models of glucocorticoid-induced osteonecrosis, underscoring the translational potential of EV-based approaches in ischemic bone disease [50]. However, EV signaling under glucocorticoid stress is not uniformly protective; exosomes from glucocorticoid-stimulated M1 macrophages promote adipogenic differentiation of BMSCs, indicating a potential pathogenic role in marrow niche remodeling [5]. Other studies show that endothelial-, mesenchymal-, and immune cell-derived exosomes mitigate steroid-induced osteonecrosis by enhancing osteoblast survival, osteogenesis, and angiogenic capacity, often through microRNA-dependent mechanisms. Together, these findings position EV-mediated signaling as a critical and context-dependent interface between Wnt regulation, glucocorticoid injury, and skeletal remodeling, with both reparative and pathogenic potential [5,51–53].

## Context matters: Biological and experimental modifiers of glucocorticoid effects on bone

In summary, the skeletal consequences of glucocorticoid excess reflect a complex interplay of direct receptor-mediated actions in osteoblasts, osteocytes, and osteoclasts, and indirect effects transmitted through BMAd, endothelial and immune cells, BMSC, and EV-mediated signaling networks. Suppression of canonical Wnt/β-catenin signaling emerges as a central mechanism linking glucocorticoid excess to impaired osteogenesis and marrow adiposity, and represents a compelling target for therapeutic intervention. Indeed, multiple pharmacological and plant-derived compounds that activate Wnt signaling, neutralize sclerostin, or modulate downstream apoptotic and cytokine pathways have shown efficacy in preclinical models of glucocorticoid-induced osteoporosis and osteonecrosis, with some agents advancing toward clinical application (Table 1). Nonetheless, a critical cautionary note must be raised regarding the many biological and environmental variables known to interact with glucocorticoid signaling in bone but which are insufficiently considered or reported in the literature. Circulating regulators may also modify glucocorticoid effects on bone: inhibition of FGF23 attenuates steroid-induced osteonecrosis, whereas GDF11 promotes recovery through pro-angiogenic mechanisms [6,7]. Sympathetic nervous system tone represents one underappreciated modulator: glucocorticoid-induced impairment of hypothalamic sympathetic outflow has been shown to propagate endothelial dysfunction and osteonecrosis [54], yet autonomic tone is rarely measured or controlled in skeletal studies, despite being affected by environmental factors, including the mild cold stress of being housed at room temperature. Sex is a fundamental variable, as glucocorticoid effects on bone marrow adipogenesis, osteoblast function, and systemic metabolism differ substantially between males and females, with androgen-glucocorticoid interactions adding further complexity [31]. Housing conditions profoundly affect basal stress physiology and glucocorticoid concentrations: temperature, humidity, the light–dark cycle, and single versus group housing are all likely to affect skeletal homeostasis to some extent, yet these parameters are inconsistently reported across studies (Tables 1 and 2). Genetic background, including mouse strain and species differences, adds additional variation in susceptibility to glucocorticoid-induced bone loss and osteonecrosis [55]. Developmental stage is equally important, as glucocorticoid actions on the growing skeleton, the adult skeleton, and the aged skeleton differ considerably in mechanism and magnitude, especially with caloric restriction. Finally, regional skeletal heterogeneity is often overlooked: trabecular outcomes measured in the proximal tibia may not reflect changes in cortical bone or in trabecula elsewhere in the skeleton [56]. Collectively, these variables represent significant sources of experimental variance and potential confounding. Greater attention to their documentation, control, and interactions with endogenous and exogenous glucocorticoids is essential if the field is to generate insights into glucocorticoid biology that can be used to combat skeletal disease.

## Figures and Tables

**Table 1:** Pharmacological interventions that affect glucocorticoid-induced bone loss and their mechanistic targets.

Reagent	Source	Glucocorticoid (GC) induced osteoporosis (GIOP)		Additional treatment	Potential Mechanism(s)	Ref.
		Animal model(s)	GC treatment			

Anti-Basigin (aBSG)	Antibody	1) Three months old male Balb/cJ mice and 2) aged (24-months) male and female C57BL/6 mice	Methylprednisolone (MPS); s.c.; 2.5 mg 21-day release pellets	aBSG; i.v.; 1 mg/kg; three times per week for 28 days	Decreases bone resorption while increasing skeletal stem cell frequency and osteogenesis	Ambrosi et al., [39]
Geraniin	Plant-derived	Six weeks old male SD rats	Dexamethasone (DEX) sodium phosphate; i.m.; 1 mg/kg; twice a week for eight weeks	Geraniin; oral gavage; 40 or 60 mg/kg; daily for eight weeks	Promotes osteoblast autophagy, proliferation, and bone via activation of the PI3K/Akt/mTOR signaling pathway	Liang et al., [57]
Pentraxin 3 (PTX3)	Recombinant protein	Twelve weeks old C57BL/6J mice	MPS; i.p.; 50 mg/kg, daily during week 1, then biweekly from weeks 2–8	Recombinate PTX3; i.p.; 200 μg/kg; twice per week for eight weeks	Activates TLR4/NF-κB signaling and suppresses FGF21, promoting osteogenesis while inhibiting pre- osteoblast apoptosis	Li et al., [10]
Phyllanthin	Plant-derived	Ten–twelve weeks old male SD rats	DEX; s.c.; 3 mg/kg; three times per week for ten weeks	Phyllanthin; oral gavage; 7.5, 15 or 30 mg/kg; *? Frequency* for ten weeks	Likely acts through modulation of the HO-1/ Nrf2 and RANK/RANKL/ OPG signaling pathways	Sun et al., [58]
Schisandrin A (SchA)	Natural compound	Two months old female Balb/c mice	DEX; i.p.; 1 mg/kg; daily for four weeks	SchA; i.p.; 25, 50 or 100 mg/kg; daily for four weeks	Activates Wnt signaling by increasing WNT5A, FZD4, β-catenin, and TCF7L1 expression, promoting osteoblast differentiation	Ai et al., [8]
Tocotrienols	Vitamin E derivative	Twelve weeks old male SD rats	DEX; i.m.; 120 μg/kg; daily for two months	Annatto tocotrienol (ATT) or palm tocotrienol (PTT); oral gavage.; 60 mg/kg; daily for two months	1) Reduces GC-induced oxidative stress, inhibiting osteoblast and osteocyte apoptosis while preventing osteoclast activation; 2) enhances BMP2/Wnt/β-catenin/Runx2 and suppresses PPARγ2 signaling pathways, promoting osteoblastogenesis and inhibiting adipogenesis	Ramli et al., [9]
**Osteonecrosis of the femoral head (ONFH)**						
Clenbuterol	Pharmaceutical	Twelve weeks old male C57BL/6J mice	MPS; i.m.; 20 mg/kg; three times per week for three weeks	Clenbuterol; s.c.; 2 mg/ kg; every other day for six weeks (after MPS treatment for three weeks)	Adrb2 agonist that mimics sympathetic outflow and promotes H-type vessel angiogenesis coupled with osteogenesis	Shao et al., [54]
Madopar	Pharmaceutical	Twenty weeks old male SD rats	LPS; i.p.; 2 mg/kg; once daily for three days; followed by MPS; i.m.; 40 mg/kg; daily for six days	Madopar; oral gavage; 0.2 g/kg, daily for six weeks	1) Increases the dopamine (DA) content; 2) DA signals through dopamine D1 receptor to attenuate GC-induced osteogenic inhibition and apoptosis	Zheng et al., [59]
Isovitexin	Plant-derived	Ten weeks old male SD rats	LPS; i.v.; 2 mg/kg; once on day 1; followed by MPS; i.m.; 30 mg/kg; daily for three days	Isovitexin; i.p.; 15 mg/ kg; every other day for four weeks	Upregulates SIRT3, inhibits excessive mitophagy, restores mitochondrial homeostasis, and reduces ferroptosis in osteoblasts	Fan et al., [60]
Jintiange (JTG)	Traditional Chinese medicine	Eight weeks old female SD rats	LPS; i.p.; 0.2 mg/kg on day 1; followed by MPS; i.m.; 100 mg/kg; once daily for three days; then 40 mg/kg; three times per week for five weeks	JTG; oral gavage; 360 mg/kg; every other day for five weeks	Inhibits osteocytes apoptosis, reduces osteoclasts number, and enhances bone mineralization	Xu et al., [11]
RU486	Biochemical inhibitor	Twelve weeks old male C57BL/6J mice	MPS; i.m.; 20 mg/kg; three times per week for three weeks	RU486; PVN injection; 1 mg/mL (*? Volume*); every other day for six weeks (after MPS treatment for three weeks)	GR inhibitor that stimulates sympathetic outflow and promotes H-type vessel angiogenesis and osteogenesis in the femoral head	Shao et al., [54]

GR, glucocorticoid receptor.

**Table 2:** Effects of genetic alteration of glucocorticoid signaling on bone. Other independent variables such as diet, aging, fracture or exogenous glucocorticoids are listed. Environment in which experiments were performed is indicated, but is often not reported.

Genetic model	Target cell(s)	Experimental condition(s)	Husbandry	Outcome(s)	Potential mechanism(s)	Ref.

**C57BL/6J**	Global	GC treatment (male and female)	8°C-23 °C; 40%–60% humidity; 12 h light-12 h dark cycle; single/group housing not specified	Trabecular bone loss due to reduced bone turnover	Impaired fatty acid transportation	Li et al., [32]
	Global	GC treatment and fracture (male and female)	8°C-23 °C; 40%–60% humidity; 12 h light-12 h dark cycle; single/group housing not specified	Inhibited callus formation and delayed fracture healing	Dysregulated macrophage-associated immune milieu contributing to osteogenesis	Li et al., [32]
**GR (*Nr3c1*)^−/−^**	Global	Fracture (male)	23 °C; 55 ± 10% humidity; 14 h light–10 h dark cycle; group housing (≤5 per cage)	Impaired fracture healing	Disruption of endochondral ossification (cartilage-to-bone transformation)	Rapp et al., [61]
	Osterix^+^ progenitors	Ad libitum or caloric restriction (female)	12 h light-12 h dark cycle; temperature, humidity and single/ group housing not specified	Reduced bone mass and increased marrow fat under ad libitum feeding	Impaired bone formation; GR is not required for CR-induced marrow adipogenesis	Pierce et al., [21]
	Osterix^+^ progenitors	Aging (female; 21 mo)	12 h light-12 h dark cycle; group housing, temperature and humidity not specified	Low bone mass and increased BMAT	Reduced mineralizing surface and aberrant marrow adipogenesis	Pierce et al., [22]
	Runx2^+^ osteoblasts	Baseline (female; 10 wk)	Husbandry information not specified	Reduced baseline bone mass; resistant to GC-induced bone loss	Impaired differentiation toward functional osteoblasts rather than reduced osteoblast numbers	Rauch et al. [19]
	Runx2^+^ osteoblasts	GR agonist-prednisolone (female)	Husbandry information not specified	Protection from GC-induced suppression of bone formation	GCs inhibit cytokines independent of GR dimerization	Rauch et al., [19]
	BMAds	Caloric restriction (male and female)	12 h light-12 h dark cycle; single housing; temperature and humidity not specified	No significant effects on bone or BMAT expansion following CR	To be determined	Schill et al., [28]
	LysM^+^ osteoclast precursors	Dexamethasone (in vitro and in vivo; sex not specified)	Husbandry information not specified	Protected from steroid-induced inhibition of osteoclast differentiation in vitro and bone formation in vivo	DEX arrested M-CSF activation of RhoA, Rac, and Vav3, which regulate osteoclast cytoskeleton	Kim et al., [24]
**11β-HSD1 (*Hsd11b1*)^−/−^**	Global	Caloric restriction (male and female)	22–23 °C; 12 h light-12 h dark cycle; single housed; humidity not specified	No effects on bone; attenuated CR-induced BMAT expansion in males	To be determined. Elevated progesterone suggestive of role in BMAd biology	Lovdel et al., [16]
	Osteocalcin (*Bglap*)^+^ osteoblasts	High fat diet (HFD; male)	12 h light-12 h dark cycle; temperature, humidity, and group/ single housing not specified	Resistant to HFD-induced trabecular bone loss and systemic metabolic disorders	Restored osteogenic activity and glucose uptake in osteoblasts	Zhong et al., [23]
**11β-HSD2 (*Hsd11b2*) OE**	Osteocalcin (*Bglap*)^+^ osteoblasts	Aging or glucocorticoid (male)	Husbandry information not specified	Prevented osteocyte apoptosis and preserved bone strength	Excess glucocorticoids induce apoptosis of osteoblasts and osteocytes	O’Brien et al., [15]
	Osteocalcin (*Bglap*)^+^ osteoblasts	Aging (21 mo; male and female)	20 °C; humidity 48%; 12 h light-12 h dark cycle, single housing	Prevented age-related loss of bone mass and strength	Reduced osteoblast and osteocyte apoptosis; improved bone microarchitecture, crystallinity and vasculature	Weinstein et al., [17]
	Col1a1^+^ osteoblasts	Aging (male and female)	24 °C; 12 h light-12 h dark cycle; humidity and group/single housing not specified	Mild reduction in trabecular bone; protection from hyperphagia, obesity and insulin resistance	To be determined	Henneicke et al., [20]
	Col2.3^+^ osteoblasts	Corticosterone (male)	Group housing (≤5 per cage); temperature, humidity, and light/dark cycle not specified	Prevented insulin resistance, glucose intolerance, and obesity	Attenuation of GC-mediated suppression of osteocalcin synthesis	Brennan-Speranza et al., [62]

GC, glucocorticoid; GR, glucocorticoid receptor; BMAT, bone marrow adipose tissue; DEX, dexamethason.

## Data Availability

No data was used for the research described in the article.
